# ReversFix-Technology—A Randomized Clinical Trial Investigating Laser Versus Conventional Removal of Ceramic Veneers and Single Crowns

**DOI:** 10.3390/dj14100666

**Published:** 2026-10-13

**Authors:** Sarah M. Blender, Heike Rudolph, Jens Dreyhaupt, Ralph G. Luthardt

**Affiliations:** 1 Center of Dentistry, Department of Prosthetic Dentistry, Ulm University Hospital, 89081 Ulm, Germany; 2 Institute of Epidemiology and Medical Biometry, University of Ulm, 89075 Ulm, Germany

**Keywords:** Er:YAG laser, restoration removal, laser removal, laser debonding, dental ceramics, veneers, ceramic crown, RCT, lithium disilicate

## Abstract

**Objectives:** The purpose of this prospective, randomized clinical study (RCT) was to investigate laser removal of ceramic restorations in terms of its effectiveness and potential advantages compared to conventional methods. **Methods:** In two study groups, with 20 patients each, veneers (study group V) and single crowns (study group C) made of lithium disilicate were adhesively fixed to vital, asymptomatic teeth. Following a period of wear of 7 ± 2 days, the patients were randomized into either of the two study arms (*n* = 10). In the control arm, the restorations were removed conventionally with diamond-coated rotary burs in combination with crown removal instruments. In the intervention arm, removal was performed with a pulsed Er:YAG laser (ReversFix-Technology). **Results:** In group V, all restorations were successfully removed in both study arms, with a significant time advantage observed in the intervention arm (*p* = 0.002). In group C, all ten restorations were removed conventionally, while in the intervention arm, seven restorations were removed initially and a further two after an additional week of wear; the time required for removal was comparable between the study arms. Using the laser-assisted technique, two veneers and seven crowns could also be removed completely in one piece. **Conclusions:** ReversFix-Technology can be considered an alternative to conventional methods for removing ceramic restorations. Laser removal even offers a time advantage, particularly for removing veneers. Moreover, in the case of non-destructive removal of the restorations, the option of recementation is possible.

## 1. Introduction

The removal of fixed ceramic dental restorations from teeth may be necessary for various biological, technical, or aesthetic reasons [1,2,3]. Conventional removal is performed using rotary burs and crown removal instruments, which carry a certain risk of damaging the underlying tooth structure and surrounding tissues. In most cases, the restorations need to be completely destroyed, making clinical reuse impossible [4,5,6,7].

All-ceramic restorations present additional challenges due to their typically adhesive fixation and the material’s inability to split and expand without fracturing [8,9,10]. This is particularly important for thin restorations that are adhesively bonded to the enamel, as these require complete, extensive grinding of the ceramics, for example, in the case of veneers. This can be very time-consuming and challenging for the dentist, as well as for the patient. Due to its excellent aesthetic properties, it is difficult to differentiate between the individual layers of tooth-colored ceramic, the (adhesive) bonding material, and the underlying tooth structure, which can lead to invasive damage of the underlying tooth structure [4,6].

The use of a laser offers a possible alternative. The emitted laser light passes through the translucent ceramic materials and interacts with the corresponding absorber components in the bonding material, which may include water molecules or residual monomers. This results in local ablation of the bonding material, followed by hydrodynamic ejection, which leads to local weakening of the bond [11,12,13,14,15]. If this is performed at several points on the bonded surface, it can lead to removal of the bonded ceramic. Er:YAG lasers with a wavelength of 2940 nm are particularly suitable for this purpose, as they have a maximum absorption in the range of water molecules and are therefore particularly well absorbed by hydrated biological structures [5,12,16,17,18].

Laser removal of dental restorations has so far been described primarily for the removal of orthodontic brackets and, to a smaller extent, for veneers and single crowns made of various ceramic materials [4,5,6,19,20,21]. These studies are based entirely on in vitro and ex vivo experimental test setups. Only a few case reports and one retrospective study describing clinical applications in humans have been published to date [22,23]. The focus of these publications is on determining and investigating suitable parameters for the successful and safe use of lasers regarding possible side effects. These include, for example, the investigation of appropriate laser parameters [24,25], the use of effective cooling mechanisms [16,26], changes in pulp and periodontal temperature [27,28], and possible surface changes to the restorations and tooth surfaces following laser application [29,30]. High-quality clinical studies on patients investigating the laser’s suitability and effectiveness for removing ceramic restorations are still lacking. Comparative studies evaluating conventional and laser-assisted procedures clinically are also unavailable.

The aim of this prospective, randomized clinical pilot study was to investigate the laser removal technique for various ceramic dental restorations in comparison to conventional methods in clinical application. The null hypothesis stated that there would be no differences between laser-assisted and conventional removal of ceramic veneers and single crowns in terms of the effectiveness of the technique (=removal possible), the time required for removal, and subjective pain perception during removal.

## 2. Materials and Methods

The present study is a single-center, prospective, randomized, controlled phase II pilot clinical trial (RCT) conducted at the Department of Prosthetic Dentistry, Center of Dentistry, at Ulm University Hospital. The investigations were conducted in accordance with the World Medical Association’s Declaration of Helsinki, ISO 14155 Clinical Investigation of Medical Devices, the Guidelines of Good Clinical Practice (2001/20/EC), and §23 of the German Medical Devices Act. The study was registered with the German Register of Clinical Trials (Freiburg; DRKS-ID: DRKS00016888) and the European Database on Medical Devices (EUDAMED; CIV-16-04-015244). The study design was approved by the Institutional Review Boards of the University of Ulm, Germany (approval number 144/16, 20 July 2016). Written informed consent was obtained from all patients included in the study prior to their enrollment.

### 2.1. Study Protocol

This study is a Phase II study involving the first use of laser removal of ceramic restorations in humans, divided into three study groups according to the restorations to be removed: brackets, veneers, or single crowns. This publication focuses on the evaluation of the veneers (group V) and single crowns (group C). Separate publication of the three study groups in two publications is necessary to preserve methodological quality (different materials, different study protocols) and because the research questions differ in certain aspects depending on the specific area of dentistry (orthodontics vs. prosthodontics). A formal case number calculation is not available due to a lack of clinical data. Due to the design of this Phase II study being the first human application, there were no comparable preliminary clinical studies that would have allowed for a formal sample size calculation. The study was therefore designed as a pilot study with *n* = 20 per study group, and thus *n* = 10 for each study arm. This sample size is based on methodological recommendations for pilot studies, enabling a feasibility analysis.

In the study groups, 20 study teeth each were restored with a ceramic veneer or a ceramic single crown made of lithium disilicate (Emax Press, HT, A3, Ivoclar Vivadent, Schaan, Liechtenstein) using an adhesive bonding material (Bifix QM, VOCO GmbH, Cuxhaven, Germany) in accordance with the manufacturer’s instructions and were removed conventionally (control; *n* = 10 per study arm) or using an Er:YAG laser (intervention; *n* = 10 per study arm). Patients with at least one vital tooth requiring extraction were recruited for this study. The teeth had to meet the clinical criteria for treatment with a ceramic veneer or a single crown. Table 1 provides a detailed overview of the inclusion and exclusion criteria for the study.

Each patient could only participate once in each study group. Patients with multiple teeth who met the inclusion and exclusion criteria were able to participate in all study groups consecutively. The recruitment of patients into the individual study groups was risk-adapted, determined by the size of the bonding surface of the restorations and the expected number of laser pulses required for removal (Brackets < Veneers < Crowns). Recruitment to the next study group only took place after half of all patients in the intervention group (*n* = 5) had been treated and the safety of the laser removal method had been evaluated. The safety review was carried out by a steering committee composed of two experts in the field of medical and dental (clinical) laser technology. In cases where a patient’s potential study tooth met the criteria for several study groups, the patient was enrolled in the study group for which recruitment was already permitted and which had the fewest patients. If several teeth of a patient were suitable for participation in a study group, they were ranked in ascending order by quadrant and tooth number according to the FDI (Fédération Dentaire Internationale) tooth scheme and assigned random numbers from a random series of numbers. The tooth that received the highest random number was defined as the study tooth.

**Figure 1:**
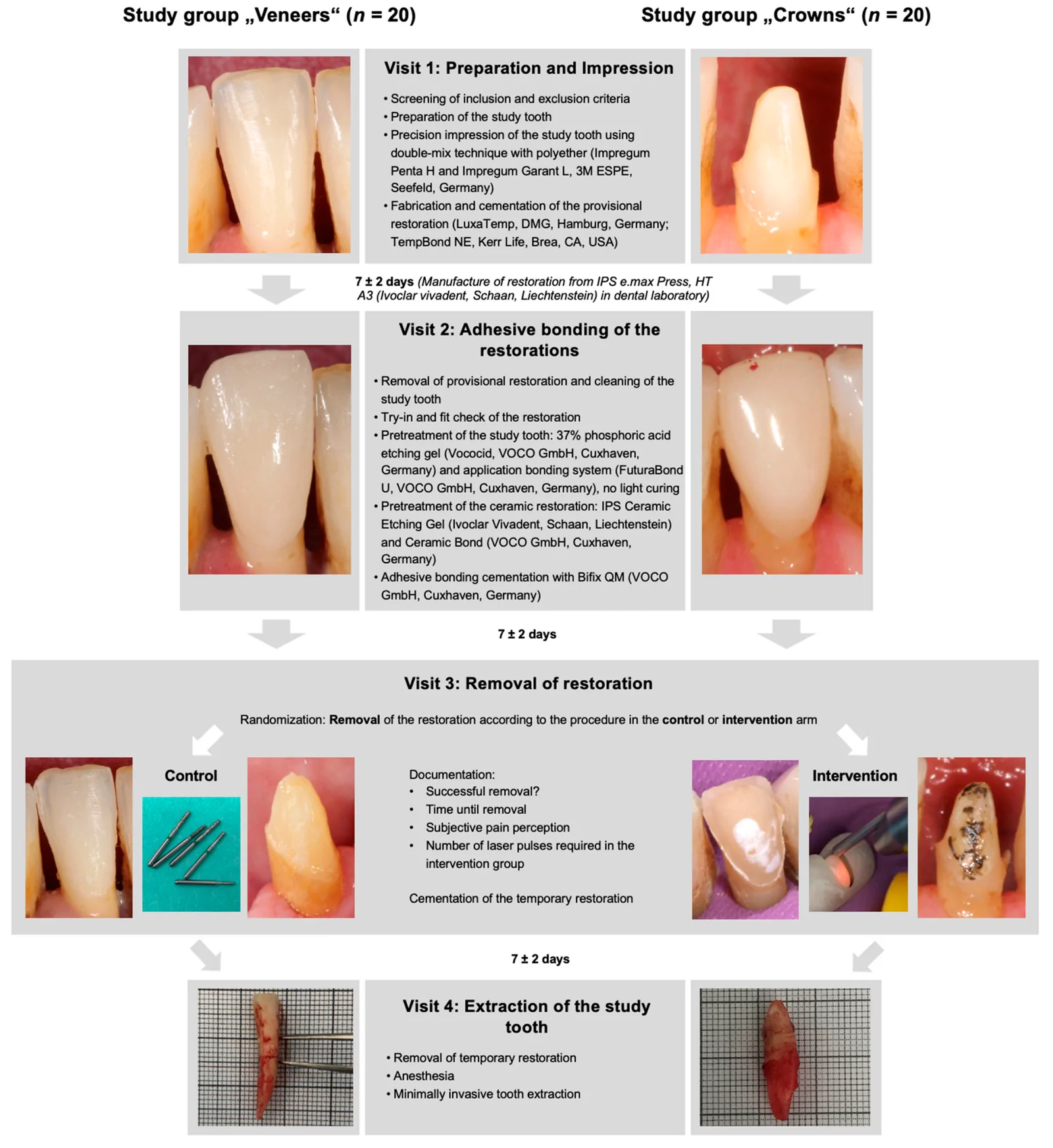
Detailed overview of the individual study visits and the treatments performed.

In both study groups, patients were treated on four visits at intervals of 7 days ±2. Figure 1 provides a detailed overview of the individual study visits with the respective study treatments performed.

### 2.2. Removal of Restorations: Conventional Versus Laser-Assisted

The ceramic restorations were removed 7 ± 2 days after cementation. Following an initial check to ensure that the restorations were intact in situ, the patients were randomized into the control or intervention arm according to the removal technique. Randomization was performed for each study group using prepared randomization envelopes from the Institute of Epidemiology and Medical Biometry at the University of Ulm. Removal was usually performed without anesthesia. However, if anesthesia was requested by the patients, it was possible at any time during removal and was documented accordingly. Conventional removal of the ceramic veneers and single crowns was performed using rotating diamond-tipped burs with water cooling in combination with crown removal instruments.

In the intervention arms, laser removal (ReversFix-Technology) was performed using a pulsed Er:YAG laser (Dermablate MCL 31, Asclepion, Jena, Germany) and an output energy of 2.03–2.05 J (fluence 30 J/cm^2^, pulse length 600 μs, repetition rate 1 Hz, spot diameter 3 mm, distance 10 mm) and air cooling with two air blowers and a large saliva aspirator. The laser pulses were applied according to a defined pattern to distribute the application over as large an area of the bonding surface as possible. After irradiating one bonding surface, a crown removal instrument (GC Pliers, GC Corporation, Tokyo, Japan) was used to carefully check whether detachment had already occurred. To protect the surrounding anatomical structures, a rubber dam was applied, and a radiation protection splint made of light-curing plastic (HS light-curing base plates, pink, Henry Schein Dental Deutschland GmbH, Langen, Germany) was placed. For safety reasons, laser removal was stopped due to the study protocol and documented as unsuccessful for the following reasons:

Criterion 1: Reaching the predefined maximum number of laser pulses (group V: 100 pulses; group C: 350 pulses).

Criterion 2: All bonding surfaces were treated four times with laser pulse removal (group V: vestibular and incisal; group C: vestibular, oral, and occlusal).

### 2.3. Study Objectives

To determine the effectiveness of the removal technique, i.e., whether removal of the restoration was possible using the corresponding technique, it was documented whether removal had taken place. In addition, it was noted whether the restoration was removed entirely or in several pieces.

To determine the time required for successful removal of the restorations, the start and end times of the removal process were documented in minutes. In the intervention arm, the number of laser pulses required for successful removal was also documented.

To determine the subjective pain intensity at the time of removal, the patient completed a visual analog scale with a length of 100 mm (0 = no pain; 100 = most severe pain imaginable). The patient was asked to document any pain sensation with a vertical line on the horizontal pain scale according to their subjective perception. The evaluation was carried out by two independent raters at two different points in time, six weeks apart. The raters did not clinically perform the removal.

### 2.4. Statistical Analysis

The existing number of cases, with 10 subjects per study arm, allows for exploratory data analysis. The demographic evaluation of the patients’ characteristics and the examined objectives used descriptive statistics. Categorical variables are described with absolute frequencies and percentages. Continuous variables are described as mean and standard deviation or median and range as appropriate. Due to a lack of normal distribution (Shapiro–Wilk test), comparisons between the control and intervention arms within each study group were analyzed for statistical significance using an appropriate nonparametric test (Mann–Whitney U test). No statistical comparison was performed between study groups “Veneers” and “Crowns.” The significance level was set at α = 0.05 (two-sided).

To determine the subjective pain intensity at the time of removal, the data recorded by the patients on the visual analog scale were assessed by both raters. Inter-rater reliability was evaluated using the intraclass correlation coefficient (ICC), based on a two-way mixed-effects model with absolute agreement for single measures (95% confidence interval). Cronbach’s alpha was also calculated to assess internal consistency.

All statistical analyses were performed using SAS, version 9.4 (The SAS Institute, Cary, NC, USA), and IBM SPSS Statistics, version 31.0.1.0 (IBM Corp., Armonk, NY, USA). Due to the exploratory nature of this study, no adjustment for multiple testing was made. All results from statistical tests must be interpreted as hypothesis-generating rather than confirmatory.

## 3. Results

Data from 20 study teeth per study group could be used for the evaluation.

### 3.1. Study Population

**Figure 2:**
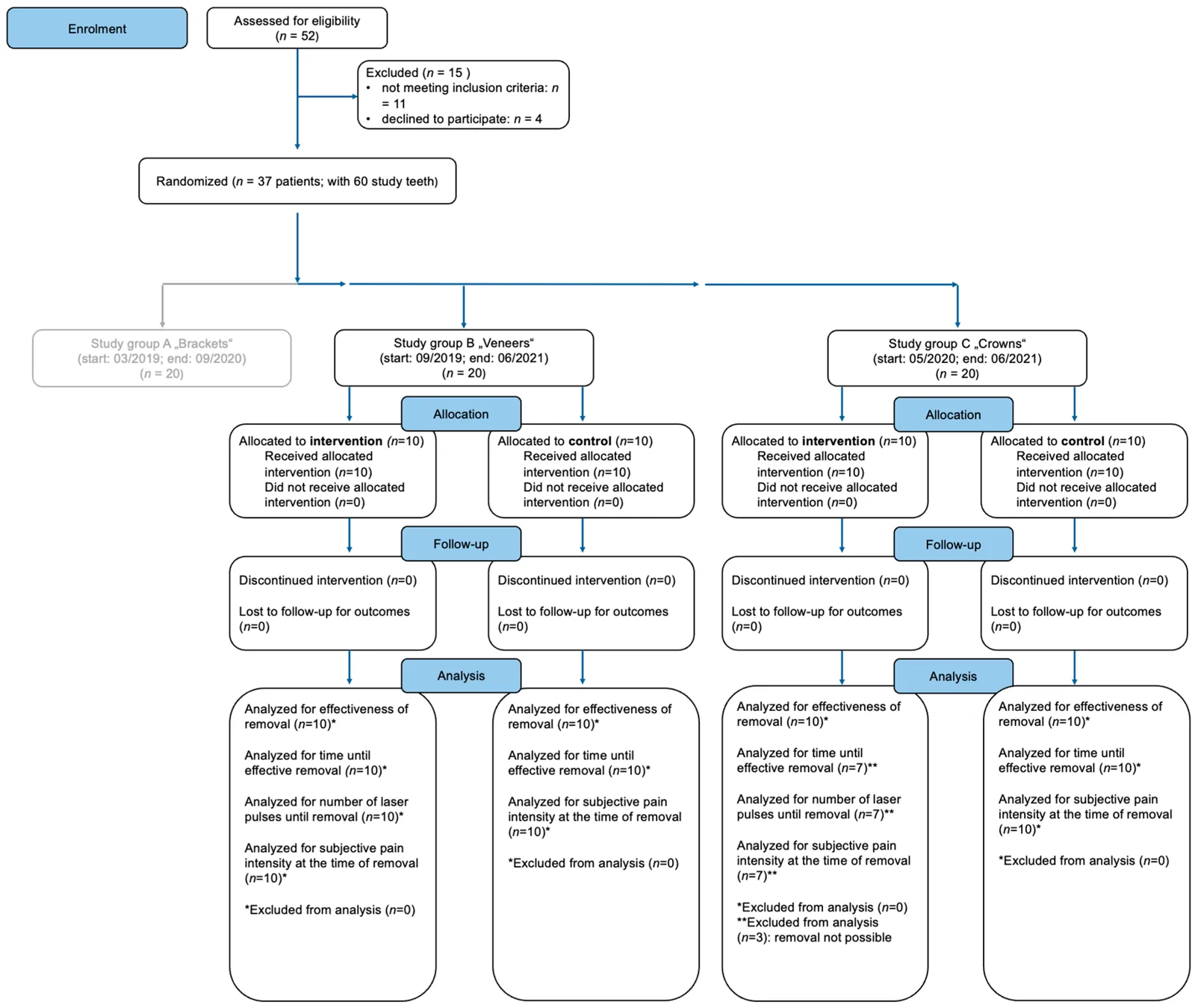
CONSORT flow diagram of the study.

Figure 2 shows the CONSORT diagram, which visualizes the entire progress of the study participants within the clinical study and the periods for risk-adapted recruitment into the individual subgroups. A total of 52 patients were screened, of whom 37 patients with a total of 60 study teeth were included. In each study group, 20 individual patients were examined, each with one tooth. Twenty patients participated in one study arm, eleven patients in two study arms, and six patients in each of the three study arms.

Table 2 provides detailed information on the study population in terms of the age and sex of the patients.

**Figure 3:**
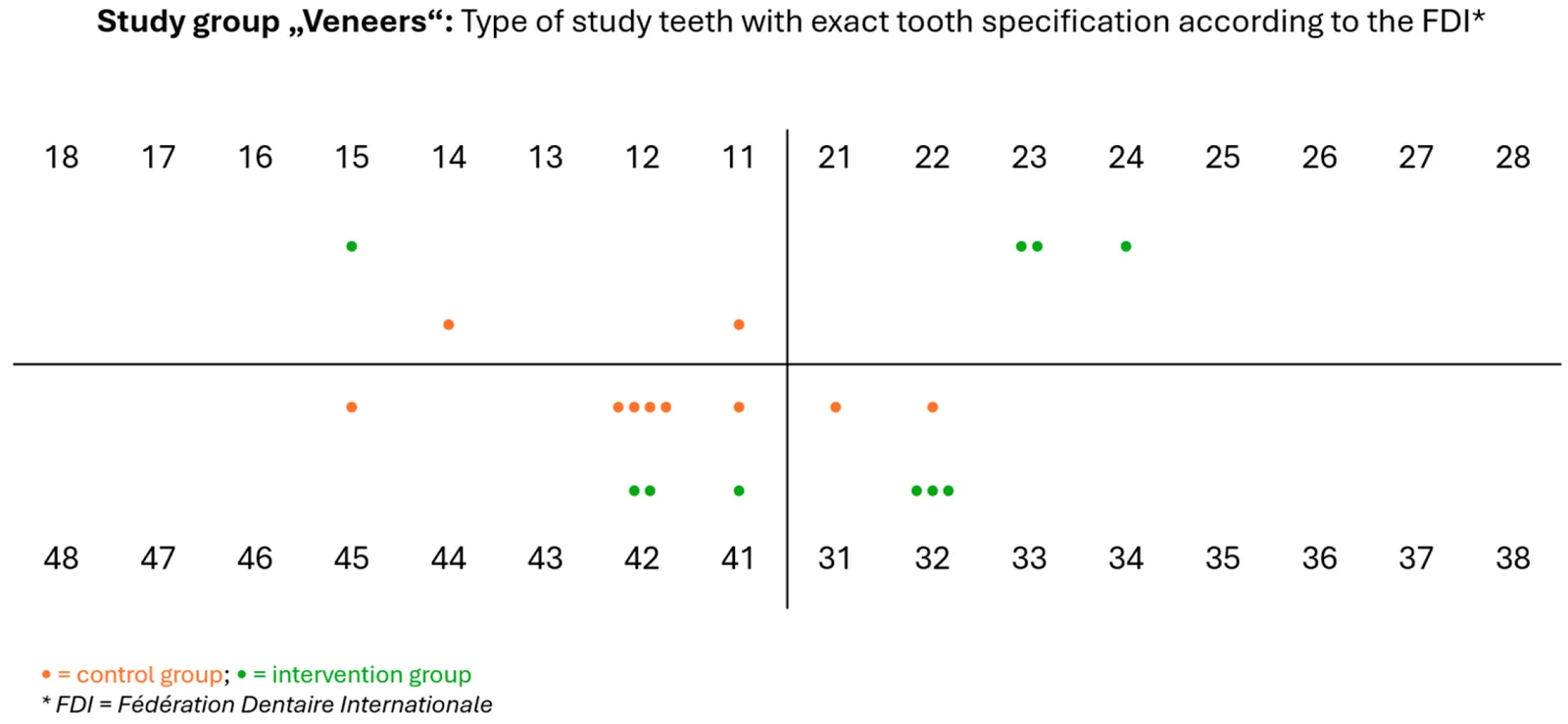
Distribution of the types of teeth used in the control and intervention arms in the study group “Veneers”.

Figure 3 shows an overview of the types of study teeth with exact tooth specifications according to the FDI scheme for the control and intervention arms of study group V.

**Figure 4:**
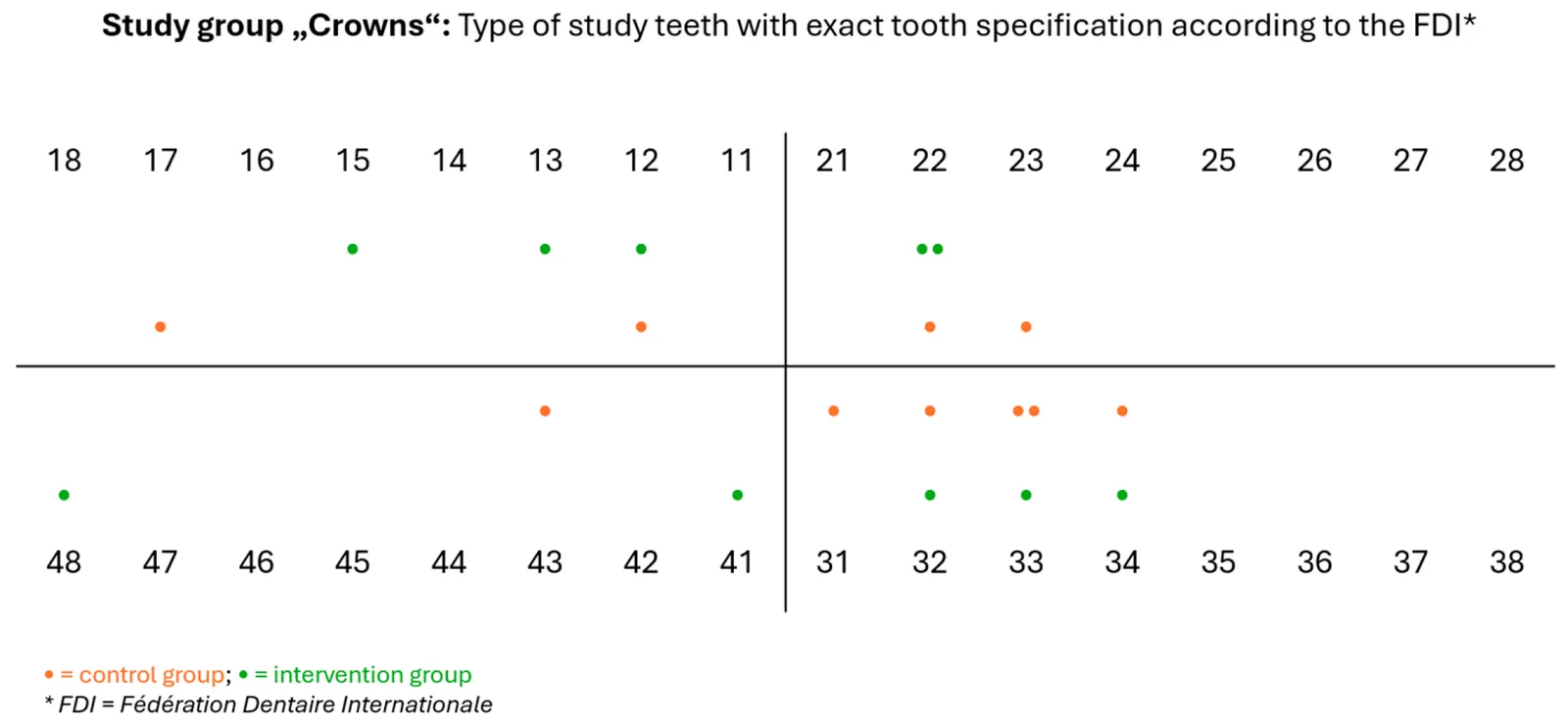
Distribution of the types of teeth used in the control and intervention arms in the study group “Crowns”.

Figure 4 shows an overview of the type of study teeth with exact tooth specifications according to the FDI scheme for the control and intervention arms of study group C.

### 3.2. Effectiveness of Removal Techniques

In study group V, all ten veneers could be removed in both the conventional and interventional groups. In study group C, ten crowns were completely removed using conventional methods, while a total of seven of the ten crowns were removed using laser-assisted technology. Two additional crowns were completely removed in advance at the time of extraction of the study tooth (visit 4). Table 3 provides an overview of the effectiveness of the removal technique and the condition of the restorations after removal.

### 3.3. Time Until Effective Removal: Conventional Versus Laser

Time required to remove the restorations using the two removal techniques is shown in Table 4. Here, only those restorations were considered where successful removal was possible. Consequently, in the group of crowns, the number of cases involving laser-assisted removal is seven. In study group on veneers, the veneers could be removed significantly faster using a laser (*p* = 0.002). No significant difference was observed in study group C.

The procedural times for the three unsuccessful laser attempts were 53, 24, and 33 min (median, 33 min; range, 24–53 min).

### 3.4. Number of Laser Pulses Until Laser Removal

All ten veneers in the intervention arm could be removed. Between 1 and 14 laser pulses were required, with a median value of 6 (IQR: 3–11).

For the seven crowns that were initially removed, between 7 and 273 laser pulses were required, with a median of 92 (IQR: 50–102) laser pulses. Laser removal was stopped once for criterion 1 (350 laser pulses) and twice for criterion 2 (267 and 292 laser pulses).

### 3.5. Subjective Pain Intensity at the Time of Removal

The measurements across two raters and two time points showed perfect agreement: ICC(2, 1) = 1.00 (95% CI [1.00–1.00]).

Table 5 also provides an overview of the documented subjective pain intensity at the time of removal within the individual study groups for all participants who experienced a successful removal. Therefore, for the group of laser-assisted crown removal, only data from seven patients could be included in the analysis.

During the removal procedure, a total of 9 patients subsequently requested anesthesia. In study group V, two patients requested anesthesia during conventional removal and one during laser-assisted removal, while in study group C, four patients requested anesthesia during conventional removal and two during laser-assisted removal. As anesthesia may influence the subjective perception of pain, its use was considered in the analysis. Accordingly, Table 6 presents the subjective pain intensity at the time of removal specifically for patients who did not receive anesthesia during the procedure.

No significant differences between the removal techniques could be documented, regardless of whether an anesthetic was used or not.

## 4. Discussion

The present study is the first to investigate the effectiveness of laser removal techniques in humans under controlled, randomized clinical conditions in comparison to conventional removal methods. The null hypothesis stating that there are no differences between laser-assisted and conventional removal of ceramic veneers and single crowns regarding removal effectiveness, removal time, and subjective pain perception was not supported for all investigated parameters. A statistically significant difference was detected for veneer removal time, whereas no statistically significant difference was detected for crown removal time. Differences were also observed, particularly in the proportion of restorations successfully removed, with these differences varying between the study groups.

Due to a lack of clinical data and the resulting inability to perform a sample size calculation, a sample size of *n* = 20 was selected for each study group, since this is sufficient to observe relative side effects and detect relevant differences between treatment methods. The study was designed as a pilot study intended to serve as proof of principle, allowing for exploratory data collection. For safety reasons, early Phase II studies typically include only a small number of patients.

In the study group of veneers, all restorations could be removed, with the laser removal technique offering a significant time advantage over the conventional technique. This can be attributed to the fact that conventional removal is only possible by completely grinding down the entire bonding surface of the restoration, due to the thin layers of ceramic. The barely visible color differences between the ceramic, bonding material, and tooth surface require a very careful and time-consuming approach to protect the underlying tooth structure [31]. Laser removal enables rapid detachment with just a few pulses. The thin layers of ceramic and the resulting high transmission of laser light [13,24,32,33], as well as the direct irradiation of the entire composite surface due to the large spot diameter of the laser (3 mm), are advantageous. The non-retentive preparation form also makes it easy to quickly detect a weakened bond.

Both conventional and laser removal of ceramic crowns required longer removal times descriptively, although no difference between the arms was observed. This is mainly due to the greater thickness of crowns and their larger bonding surface. In conventional removal, more material must be separated with rotating burs at different points to preserve the underlying tooth structure. With laser-assisted technique, the transmission behavior of the ceramics is crucial, as increasing thickness reduces laser transmission, and less energy reaches the absorber [6,11,13,34,35]. Additionally, bonding must be addressed on multiple surfaces, not only vestibularly. However, adjacent teeth often prevent laser access to approximal surfaces. Some in vitro studies do not consider this clinical limitation, which may influence their results [15,27,36].

The retentive preparation geometry of crowns significantly affects removal time, particularly with laser techniques. Unlike veneers, crowns do not show a sudden release, as the wedging effect of the bonded surfaces keeps them in place even when the bond is weakened. Micro-explosions during bond disruption create roughness on the surface, further increasing retention. Preparations with small angles, parallel walls, and high axial walls are especially challenging. Regular checks between laser applications are therefore required, prolonging removal time. In this study, crown pliers with rubber nubs were used to prevent ceramic damage. However, a standardized removal force cannot be defined, as it varies between practitioners.

Initially, seven crowns were removed within the study criteria; the remaining three might have required additional laser pulses or greater force. The right combination of various parameters, such as layer thickness and laser settings, appears to be crucial in this regard [11,36]. Laser treatment appears to weaken the adhesive bond [5,27], as two crowns were successfully removed after another week of function. Clinically, even delayed removal of intact restorations can be considered a success. Comparison with previous studies is limited, as available data are based solely on in vitro or ex vivo designs [5,6,37]. Whilst the removal of the veneer occurred at a comparable rate or slightly faster [24,26,34,38,39,40], crown removal was more time-consuming in this study for both the intervention [15,27,41,42] and control groups [25]. In addition to ceramic thickness and laser parameters, this is mainly due to clinical factors such as patient positioning, laser handling, access to bonding surfaces, and verification of debonding, which cannot be standardized in vitro [43,44,45]. The discrepancy between the number of laser pulses and total removal time highlights the influence of these external factors. Assuming one pulse per second (1 Hz), fewer pulses than total seconds would theoretically be required. Reporting pulse numbers therefore better reflects direct laser effectiveness, independent of procedural variables. However, as clinical conditions are crucial for therapeutic evaluation, future studies should continue to document total treatment time. Moreover, pulse-based assessment is not applicable to unpulsed lasers, limiting comparability.

This study also demonstrates the potential of laser-assisted removal technology in terms of the possibility of complete and non-destructive removal of the ceramic. While this was observed in study group V in two test samples, there were initially five crowns in study group C. If we consider the two crowns that could be removed after a further week of wear, this number rises to seven crowns. The same laser settings were used for both study groups, resulting in a very high initial output energy. While this is necessary for the removal of single crowns due to increased layer thickness, enlarged bonding surface, and preparation shape, it can lead to complete fracture of the ceramics in veneers after just a few laser pulses. For possible non-destructive removal, it can therefore be considered whether a reduction in fluence would be advantageous [28,46]. For crowns, it was observed that non-destructive removal of restorations requires a higher number of laser pulses. Accordingly, the fastest possible removal time is not always preferable, especially if reuse of the restorations is planned [43].

The intensity of pain reported by patients during removal in the control and intervention arms of the two study groups is approximately comparable. Similar to the results for the time required for removal, the subjective pain sensation reported also increases with increasing bonding surface area (veneers < single crowns), regardless of the removal technique used. Due to a lack of publications in this field, a comparison with other studies is not possible. With conventional removal, the sensitivity or pain reported is usually due to the water and air cooling used. Heat generation from rotating instruments, as well as unpleasant vibrations and forces on the tooth surface from crown removal instruments, may also be possible causes. For laser removal, this can also be explained by the cooling strategy used, as well as by possible laser-induced heat generation. Although Er:YAG lasers have a high energy potential, they are almost completely absorbed by suitable absorbers in the luting material or in the hydrated tooth structure, resulting in a very short exposure time and preventing any potential temperature development. In addition, some of the heat is removed immediately by detached particles [27,47]. In vitro and ex vivo results show that additional cooling strategies and distribution of laser pulses over a large area with continuous movements prevent pulp and periodontal temperatures from rising above the specified limits [16,18,27,36].

One limitation of the present study is that it only examined the influence of the described materials and parameters under the specified study conditions. Further studies are needed to evaluate the influence of these factors to enable safe application in patients. In addition to the different restoration materials and luting materials, the focus should also be on the laser settings used, the influence of different preparation shapes, and the design of the cement gap.

## 5. Conclusions

Based on the results of this prospective, controlled clinical study and considering the parameters used here, the following conclusions can be reached:

(1) The use of an Er:YAG laser for removing ceramic veneers and single crowns offers a comparable alternative method to conventional removal techniques.
(2) Regarding the time required to remove the restorations, the laser-assisted method offers a time advantage, especially for veneers. The time required to remove single crowns shows comparable results under the study conditions specified here.
(3) The laser removal technique offers the potential of a non-destructive alternative for removing ceramic restorations, thereby enabling their clinical reuse.

## Figures and Tables

**Table 1:** Overview of the defined inclusion and exclusion criteria for the study.

Inclusion Criteria	Exclusion Criteria
- Legally competent patient between 18 and 80 years of age - Written informed consent (at least 24 h after explanation) - Vital tooth indicated for extraction without acute pain symptoms - Intact, uninjured gingiva or tissue around the tooth - The teeth had to meet the clinical criteria for treatment with a ceramic veneer or a single crown *^,^ **	- Pregnancy or breastfeeding - Infectious diseases (viral hepatitis and HIV) - Alcohol or drug abuse - Teeth with widely spread roots on radiographs that would require sectioning prior to extraction - Teeth affected by molar-incisor hypomineralization - Teeth affected by amelogenesis imperfecta - Teeth affected by dentinogenesis imperfecta - Turner’s teeth - Human beings in a dependent relationship (employees of the Clinic for Prosthodontics and dental students at Ulm University) - Accessibility of the study tooth surface treated according to the study group not possible for laser treatment

* For the veneer study group: preparation margins after preparation must be located entirely in enamel (veneer preparation may be vestibular, oral, or, if applicable, approximal). ** For the single-crown study group, full crown preparation is possible.

**Table 2:** Overview of the data collected from the control and intervention arms in the two study groups, “Veneers” and “Crowns,” with respect to the participants’ age and sex.

	Study Group “Veneers”		Study Group “Crowns”	
	control (*n* = 10)	intervention (*n* = 10)	control (*n* = 10)	intervention (*n* = 10)
sex	female = 11, male = 9		female = 10, male = 10	
	female = 7, male = 3	female = 4, male = 6	female = 4, male = 6	female = 6, male = 4
age * (in years)	59.6 (SD ± 10.4; Max 79.0; Min 37.0)		63.2 (SD ± 8.0; Max 79.0; Min 44.0)	
	61.8 (SD ± 11.0; Max 79.0; Min 45.0)	57.4 (SD ± 9.8; Max 72.0; Min 37.0)	65.2 (SD ± 6.7; Max 74.0; Min 56.0)	61.1 (SD ± 9.0; Max 79.0; Min 44.0)

* Age at the time of intervention.

**Table 3:** Overview of the data collected from the control and intervention arms in the two study groups, “Veneers” and “Crowns,” with respect to the effective removal of the restorations.

	Study Group “Veneers”		Study Group “Crowns”	
	Control (*n* = 10)	Intervention (*n* = 10)	Control (*n* = 10)	Intervention (*n* = 10)
removed	10	10	10	7 *
restorations complete after removal	0	2	0	5 *

* Two additional crowns could be removed completely directly before extraction of the study tooth (7 ± 2 days after removal).

**Table 4:** Overview of the data collected from the control and intervention arms in the two study groups, “Veneers” and “Crowns,” with respect to time until effective restoration removal (min).

	Study Group “Veneers”		Study Group “Crowns”	
	Control (*n* = 10)	Intervention (*n* = 10)	Control (*n* = 10)	Intervention (*n* = 7)
Mean ± SD	12.00 ± 5.14	4.50 ± 4.43	15.90 ± 4.75	17.57 ± 9.43
Median (Q1–Q3)	11.50 (7.75–14.75)	3.00 (1.00–6.75)	15.00 (12.75–21.00)	17.00 (6.00–26.00)
Min–Max	7.00–23.00	1.00–13.00	9.00–22.00	6.00–31.00

**Table 5:** Overview of the data collected from the control and intervention arms in the two study groups, “Veneers” and “Crowns”, with respect to subjective pain intensity at the time of removal.

	Study Group “Veneers”		Study Group “Crowns”	
	Control (*n* = 10)	Intervention (*n* = 10)	Control (*n* = 10)	Intervention (*n* = 7)
	*Subjective pain intensity at the time of removal, regardless of anesthesia*			
Mean ± SD	1.95 ± 3.45	7.50 ± 23.71	13.15 ± 18.13	22.75 ± 32.87
Median (Q1–Q3)	0.22 (0.00–3.72)	0.00 (0.00–0.00)	4.06 (0.00–26.95)	1.42 (0.00–63.00)
Min–Max	0.00–10.81	0.00–74.97	0.00–48.66	0.00–76.02

**Table 6:** Overview of the data collected from the control and intervention arms in the two study groups, “Veneers” and “Crowns,” regarding subjective pain intensity at the time of removal. Only patients who did not request anesthesia during the removal procedure were included in the analysis.

	Study Group “Veneers”		Study Group “Crowns”	
	Control (*n* = 8)	Intervention (*n* = 9)	Control (*n* = 6)	Intervention (*n* = 5)
	*Subjective pain intensity at the time of removal depending on anesthesia*			
Mean ± SD	0.99 ± 1.70	0.00 ± 0.00	9.07 ± 19.53	4.05 ± 8.28
Median (Q1–Q3)	0.00 (0.00–2.89)	0.00 (0.00–0.00)	0.00 (0.00–16.46)	0.00 (0.00–10.12)
Min–Max	0.00–3.74	0.00–0.00	0.00–48.66	0.00–18.82

## Data Availability

The data supporting the findings of this study are not publicly available due to ethical and data protection restrictions related to participant confidentiality. Confidentiality-protected data may be available from the corresponding author upon reasonable request and subject to approval by the relevant ethics committee.

## Abbreviations

The following abbreviations are used in this manuscript:

RCT	Randomized Controlled Trial
C	The Crown Study Group
V	The Veneer Study Group
B	The Bracket Study Group
Er:YAG	Erbium–Yttrium–Aluminum–Garnet Laser
HT	High Translucency
HIV	Human Immunodeficiency Virus
FDI	Fédération Dentaire Internationale → English: World Dental Federation
Q1	First Quartile
Q3	Third Quartile
IQR	Interquartile Range
ICC	Intraclass Correlation Coefficient
CI	Confidence Interval
BMBF	Bundesministerium für Bildung und Forschung → English: Federal Ministry of Education and Research
